# The Effect of Coffee Consumption on the Prevalence of Diabetes Mellitus: The 2012–2016 Korea National Health and Nutrition Examination Survey

**DOI:** 10.3390/nu11102377

**Published:** 2019-10-05

**Authors:** Yejee Lim, Youngmi Park, Sun Kyu Choi, Soyeon Ahn, Jung Hun Ohn

**Affiliations:** 1 Division of General Internal Medicine, Department of Internal Medicine, Seoul National University Bundang Hospital, 82, Gumi-ro 173-beon-gil, Bundang-gu, Seongnam-si, Gyeonggi-do 13620, Korea; jhohn2@gmail.com; 2 Medical Research Collaborating Center, Seoul National University Bundang Hospital, 82, Gumi-ro 173-beon-gil, Bundang-gu, Seongnam-si, Gyeonggi-do 13620, Korea; 02141@snubh.org (Y.P.); crosspos@snubh.org (S.K.C.); ahnsoyeon@gmail.com (S.A.)

**Keywords:** coffee, diabetes mellitus, prevalence, Korea

## Abstract

An inverse association between coffee consumption and the risk of diabetes mellitus (DM) has been observed. However, little is known about this association in Koreans, although they are now among the top global consumers of coffee. Therefore, the aim of this study was to evaluate the association between the prevalence of DM and the amount of coffee consumption using a unit of exact measurement, regardless of the type of coffee consumed. This study was based on data acquired from the Korea National Health and Nutrition Examination Survey 2012–2016. The participants who completed the survey were included in the statistical analysis (*n* = 14,578). Subjects were stratified by age (19–39 years old: young adult; 40–64 years old: middle-aged adult) and gender (men, women). The amount of coffee was measured using a teaspoon (tsp) unit corresponding to 5 mL of powdered coffee and was analyzed as a continuous variable. The mean powdered coffee intake per day was 1.97 tsp in women groups, 2.24 tsp in young adult men, and 2.72 tsp in middle-aged men. The frequency of coffee consumption showed an inverse relationship with the amount of coffee intake at a time. With each 1-tsp increment in daily coffee intake, the odds of DM were 0.89 (95% confidence interval (CI): 0.86–0.92, *p* < 0.001) and 0.93 (95% CI: 0.90–0.95, *p* = 0.003) in middle-aged women and men, respectively. Coffee consumption was inversely correlated with the prevalence of DM even with adjustment for covariates in middle-aged adults. We delineated that the prevalence for DM decreased as coffee intake increased in Korean middle-aged adults. Therefore, our data represented an inverse association between coffee consumption and the prevalence of DM, although Koreans have a unique coffee-drinking habit.

## 1. Introduction

Coffee is one of the most frequently consumed beverages worldwide. Recent data have shown that Koreans drink coffee approximately 11.3 times per week, making coffee the most frequently consumed food or beverage item, followed by kimchi (9.7 times/week) and multigrain rice (8.1 times/week) [1]. Korea’s coffee industry has grown in recent years to become the 11th-largest coffee market in the world. According to an industry survey, Korean adults over 20 years of age drank 592 cups in 2014, which was an average of 1.6 cups per day. Coffee is preferred to tea in respect to hot drinks in South Korea [2].

The prevalence of type 2 diabetes mellitus (DM) has recently been increasing in Asian populations, including Koreans, reaching levels similar to those in Western countries [3]. According to the recently published report of the Korea National Health and Nutritional Examination Survey (KNHANES), the prevalence of diabetes was 10.6% and 13.0% in 2015 and 2016 respectively, among Koreans aged 30 years or older [1]. Given the substantial burden of DM, much attention has been given to the developing nutritional approaches to prevent the development of DM or to alleviate its complications. Due to the broad consumption of coffee, numerous studies have examined the effect of coffee intake on the development of DM. Previous studies showed coffee intake might be inversely associated with DM incidence and this association was not modified by the adjustment of behavioral and dietary factors [4,5].

Filtered coffee is the most common type of coffee consumed in Europe and the United States, where the majority of the previous studies were conducted [4,5]. However, instant coffee comprises the highest proportion of the coffee market share in Korea [2]. In addition, most of the previous studies analyzed coffee amount only using frequency of intake, although serving size for coffee and the amount of consumption at a time can differ substantially within and between countries and individuals. Furthermore, little data on the Korean population have been published. Therefore, the aim of this study was to evaluate the association between the prevalence of DM and the amount of coffee consumption, using a unit of an exact measurement, regardless of the type of coffee consumed.

## 2. Materials and Methods

### 2.1. Data Collection

This study was based on data acquired from the KNHANES, which has been periodically conducted by the Division of Health and Nutritional Survey in the Korean Centers for Disease Control and Prevention (KCDCP) since 1998. It is an ongoing, population-based, cross-sectional, and nationally representative survey, and it provides the largest publicly available database in South Korea. Participants were chosen from candidates via proportional-allocation systematic sampling with multistage stratification (by age, gender, and region). Twenty households from 192 randomly selected sampling units were chosen in 2012–2015, whereas 23 households from 192 sampling units were chosen in 2016. The KNHANES consists of three different components: a health interview, a nutrition survey, and a health examination. The nutrition survey incorporates three methods: a food frequency questionnaire, a dietary intake one (24 h recall method), and dietary habits research (research on items related to dietary life and eating habits) [6].

### 2.2. Subjects

**Figure 1:**
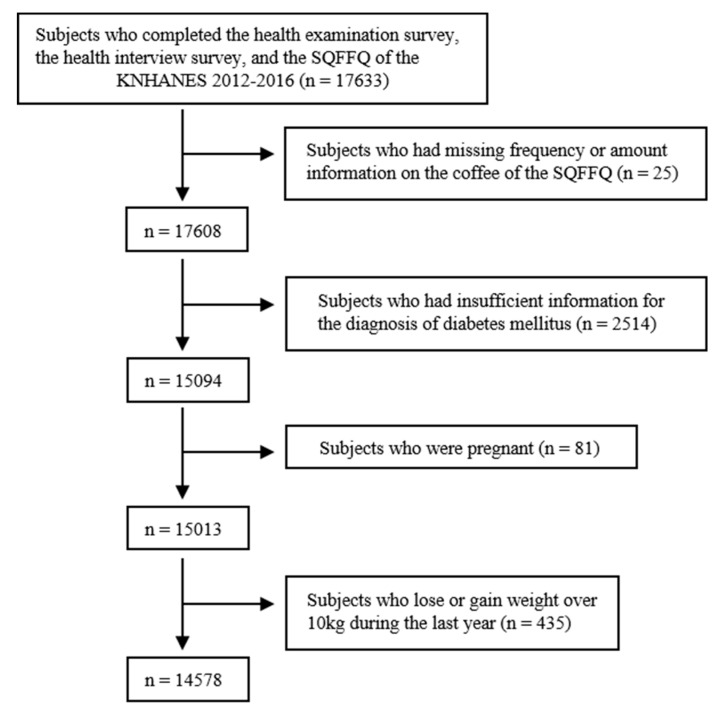
Flow diagram for the identification of the study population. A total of 14,578 subjects were finally included. KNHANES, Korea National Health and Nutrition Examination. SQFFQ, semi-quantitative food frequency questionnaires.

We combined five consecutive years’ worth of data (from 2012 to 2016). Next, we included subjects who completed the health examination survey, the health interview survey, and the food intake data collected using the validated semi-quantitative food frequency questionnaire (SQFFQ). During the study period, the SQFFQ was completed by 17,633 adults aged 19–64 years. Of the participants, 3055 were excluded according to the following exclusion criteria: (1) subjects who had missing frequencies or other data points on the coffee item of the SQFFQ (25 subjects), (2) subjects who had insufficient information for the diagnosis of DM (2514 subjects), (3) pregnant women (81 subjects), and (4) subjects who lost or gained weight over 10 kg during the last year (435 subjects). In summary, 14,578 participants qualified for statistical analysis (Figure 1) and the weighted number of participants for representing noninstitutionalized Korean civilization population was 26,245,415. The institutional review board of the Seoul National University Bundang Hospital approved this study (number X-1904-535-902). Because aging is a potent risk factor for DM and the association between coffee and DM was stronger for women than men in previous studies [5], subjects were stratified by age (19–39 years old: young adult; 40–64 years old: middle-aged adult) and gender (men, women). Therefore, analysis was conducted for four subgroups: young women (*N* = 5,579,373), young men (*N* = 6,130,535), middle-aged women (*N* = 7,416,029), and middle-aged men (*N* = 7,119,478).

### 2.3. Nutrition Survey

To assess the frequency of coffee consumption, we utilized the FFQ of the KNHANES. FFQ is a method of measuring usual food intake over long periods [7]. Before 2012, the frequency of consumption, including coffee, was categorized; however, the amount of intake at a time had not been assessed. The intake frequency for coffee has been divided into the following 9 categories: never or seldom, once per month, 2 to 3 times per month, once per week, 2 to 4 times per week, 5 to 6 times per week, once per day, twice per day, and 3 or more times per day. The participants indicated the frequency of their intake of each food based on the portion size definitions provided. Since 2012, the amount of coffee consumed at a time was measured using a teaspoon unit. One teaspoon of powdered coffee or additives corresponded to 5 mL of coffee or additives, respectively. In addition, KNHANES developed a manual to calculate coffee concentration among different types of coffee beverages. For example, one instant coffee mix consisted of one teaspoon of coffee, sugar, and powdered creamers. A 100 mL quantity of filtered coffee corresponded to 1 teaspoon of powdered coffee. A readymade can of coffee consisted of two teaspoons of coffee, sugar, and powder creamers. Kim et al. demonstrated that the updated SQFFQ used in the KNHANES from 2012 onward exhibited acceptable reproducibility and modest validity [8]. Daily intake was computed based on the midpoint of the assigned frequencies of each category for each food item. Using the SQFFQ food intake data, the intake of energy and nutrients was calculated using the Can-Pro 2.0 software [9]. Coffee intake was calculated by multiplying the midpoints of frequencies by the number of teaspoons consumed at any given time of intake.

### 2.4. Health Interviews and Examinations

Information regarding demographic and social factors was obtained using a standardized questionnaire during the health interview. Heavy alcohol drinking was categorized as drinking two or more times per week. Current smoking was defined as currently smoking with a smoking history of ≥100 cigarettes in a lifetime. After fasting for at least 8 h, blood samples were collected in the morning and analyzed at a central, certified laboratory. Plasma glucose, total cholesterol, high-density lipoprotein cholesterol (HDL-C), and triglyceride (TG) were measured using a Hitachi Automatic Analyzer 7600 (Hitachi, Tokyo, Japan). Glycosylated hemoglobin (HbA1c) was measured using high-performance liquid chromatography (HLC-723G7; Tosoh, Tokyo, Japan). The diagnosis of diabetes was based on fasting plasma glucose (≥126 mg/dL), the current use of antidiabetic medication, a history of previous diabetes, or glycosylated hemoglobin (HbA1c) ≥6.5%. High fasting glucose was defined by either a fasting glucose of ≥100 mg/dL or the presence of previously diagnosed diabetes [10]. The following definitions were obtained from the third report of the National Cholesterol Education Program Adult Treatment Panel III [11]. Hypertension was defined by either a self-reported history of hypertension diagnosis and current usage of antihypertensive drugs or a systolic blood pressure (BP) of ≥130 mm Hg, or a diastolic BP of ≥85 mm Hg. Hypertriglyceridemia was defined by TG of ≥150 mg/dL. Reduced high-density lipoprotein (HDL) cholesterolemia was defined by HDL <40 mg/dL for men and <50 mg/dL for women. Income level was categorized into 4 quartiles and education level was categorized into 2 groups: high school or under and college or higher. Physical activity was assessed by walking time and frequency. Physically active meant walking ≥5 days in a week for ≥30 min in a day for ≥10 min at a time.

### 2.5. Statistical Analysis

All statistical analyses were performed with STATA version 15.0 (Stata Corporation, College Station, TX, USA). The weights for each respondent, representing the inverse of their sampling probabilities, were provided by the KCDCP. We utilized complex stratified multistage probability sampling weights by using the “svy” command to obtain estimates that were representative of the noninstitutionalized Korean civilization population. All data are presented as the means ± standard errors (SEs) or numbers and percentages, unless otherwise specified. The four subgroups were compared in terms of baseline characteristics by ANOVA for continuous variables and chi-square tests for categorical variables. Linear regression analysis was used to evaluate the association between the frequency of coffee intake and the amount of coffee consumed at a time. Additionally, the association between the amount of daily coffee consumption and various covariates was investigated by using linear regression analysis. We considered two clinical outcomes, prevalent DM or high fasting glucose, to estimate the odds ratios (ORs) per tsp increment in daily coffee consumption. We fitted two models in which adjustments were made for various covariates. All reported probabilities (*p*-values) were two-sided with *p* ≥ 0.05 considered statistically significant.

## 3. Results

### 3.1. Baseline Characteristics

The participants’ baseline characteristics are presented in Table 1. Their mean age was 29.4 ± 0.2 in young women, 29.5 ± 0.2 in young men, 51.0 ± 0.1 in middle-aged women, and 50.5 ± 0.1 in middle-aged men. Their mean BMI was 21.8 ± 0.1 kg/m^2^ in young women, 24.2 ± 0.1 kg/m^2^ in young men, 23.7 ± 0.1 kg/m^2^ in middle-aged women, and 24.5 ± 0.1 kg/m^2^ in middle-aged men. Male groups were more likely than female groups to be current smokers and frequent alcohol drinkers. Fasting glucose, HbA1c, and the prevalence of hypertension were higher in middle-aged adults or males than in young adults or females. The mean fasting glucose and HbA1c measurements were 90.3 ± 0.4 mg/dL and 5.38 ± 0.0% in young women; 92.5 ± 0.3 mg/dL and 5.4 ± 0.0% in young men; 98.6 ± 0.4 mg/dL and 5.74 ± 0.0% in middle-aged women; and 104.1 ± 0.5 mg/dL and 5.84 ± 0.0% in middle-aged men. Young men and middle-aged women had the highest and the lowest total energy intakes, respectively, among groups.

### 3.2. Pattern of Coffee Consumption

The daily amount of coffee intake varied by groups: male groups had a higher mean intake than did female groups. Specifically, the mean powdered coffee intake per day was 1.97 ± 0.05 tsp in young women, 2.24 ± 0.07 tsp in young men, 1.97 ± 0.03 tsp in middle-aged women, and 2.72 ± 0.05 tsp in middle-aged men. In addition, coffee consumption frequency and amount of coffee intake at a time varied by groups. Middle-aged adult groups consumed coffee more frequently than young adult groups did. In contrast, young adult groups consumed larger amounts of coffee at a time than did middle-aged adult groups. Specifically, 80% of middle-aged men consumed just 1 tsp of coffee at a time but their coffee consumption frequency was the highest (15 times per week); 40% of young women consumed 2 tsp or more coffee at a time but they consumed coffee only occasionally (eight times per week) (Table 2). Finally, an inverse correlation was observed between coffee consumption frequency and the amount of coffee intake at a time (Table 3).

### 3.3. The Association between Coffee Intake and Covariates

Subjects who consumed more coffee were more likely to be smokers, consumed higher amounts of alcohol, had higher income, had higher educational attainment, had higher levels of blood cholesterol, and reported a greater total energy intake. Daily coffee consumption was positively correlated with age in young adult groups. In contrast, daily coffee consumption was inversely correlated with age in the middle-aged adult groups. There were inverse associations between the daily amount of coffee intake and the levels of fasting glucose concentration or HbA1c in middle-aged men and women, respectively. In contrast, no association was observed between the daily amount of coffee intake and a level of fasting glucose or HbA1c in young women. No considerable differences in the distribution of daily coffee intake by BMI were observed (Table 4).

### 3.4. The Univariate Association between the Prevalence of Diabetes and Amount of Coffee Consumption

**Figure 2:**
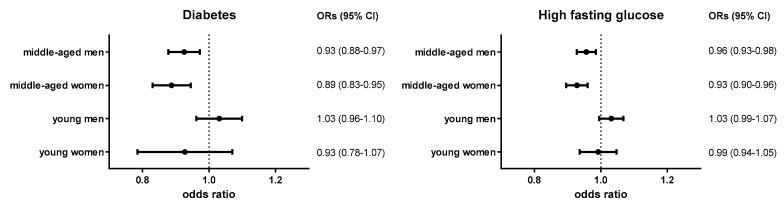
Odds ratios of diabetes and high fasting glucose according to the amount of coffee consumption.

The prevalence of diabetes was 1.90% in young women, 2.09% in young men, 9.88% in middle-aged women, and 14.92% in middle-aged men. The prevalence of high fasting glucose was 8.52% in young women, 15.68% in young men, 29.09% in middle-aged women, and 46.36% in middle-aged men. The odds of DM were 0.89 (95% confidence interval (CI): 0.83–0.95, *p* < 0.001) and 0.93 (95% CI: 0.88–0.97, *p* = 0.003) with 1-tsp increments of daily coffee intake in middle-aged women and men, respectively. In addition, the odds of high fasting glucose were 0.93 (95% CI: 0.90–0.96, *p* < 0.001) and 0.96 (95% CI: 0.93–0.98, *p* = 0.003) with 1-tsp increments in daily coffee intake in middle-aged women and men, respectively. However, the odds of DM or high fasting glucose were not significantly associated with the daily amount of coffee intake in young adult groups (Figure 2).

Diabetes: either fasting plasma glucose ≥126 mg/dL, current use of antidiabetic medication, a history of previous diabetes, or glycosylated hemoglobin (HbA1c) ≥6.5%; high fasting glucose: either fasting glucose of ≥100 mg/dL or the presence of previously diagnosed diabetes.

### 3.5. The Multivariate Association between the Prevalence of Diabetes and amount of Coffee Consumption with the Adjustment of Other Covariates

Table 5 exhibited the adjusted odds ratios for DM and high fasting glucose according to daily amount of coffee consumption after adjustment for lifestyle factors and socioeconomic status. Coffee consumption was inversely correlated with the prevalence of DM and high fasting glucose, even after adjustment for covariates. In model 1, adjustments were made for age, BMI, smoking state, and alcohol consumption. With daily 1-tsp increments in coffee intake, the odds of DM were 0.92 (95% CI: 0.85–0.99, *p* = 0.023) and 0.93 (95% CI: 0.88–0.99, *p* = 0.022) in middle-aged women and men, respectively. Even after adjustment for more potential covariates in model 2 (age, BMI, smoking state, alcohol consumption, physical activity, income level, education level, and daily energy intake), the overall trend of the inverse relationship did not change. Similar trends were observed in these models for high fasting glucose. In model 1, the odds of high fasting glucose were 0.95 (95% CI: 0.91–0.98, *p* = 0.007) and 0.96 (95% CI: 0.93–0.99, *p* = 0.018) in middle-aged women and men, respectively, with daily 1-tsp increments in coffee intake. Even after adjustment for more potential covariates in model 2, the overall trend of the inverse relationship between the prevalence of high fasting glucose and coffee intake did not change.

## 4. Discussion

The present study delineated the inverse relationship between coffee intake and the prevalence of DM using public data representing the entire Korean population. In addition, we measured the amount of coffee using an exact unit of measurement of powdered coffee. For every 1-tsp (5 mL) increase in daily coffee intake, the odds of DM were 0.89 in middle-aged women and 0.93 in middle-aged men. Even after adjustment for covariates, the inverse relationship between the prevalence of DM or high fasting glucose and the amount of coffee consumption did not change.

In the present study, the frequency of coffee consumption and the amount of coffee intake at a time showed an inverse correlation. Therefore, the examination of the frequency of coffee intake alone might make it difficult to analyze the association between coffee intake and the risk of DM. Two studies from KNHANES 2007–2011 showed that coffee consumption was not associated with fasting glucose levels or the prevalence of high fasting glucose [12,13]. This irrelevant association was shown not only in the instant coffee drinkers but also in the filtered coffee drinkers [12]. These studies, however, analyzed the intake frequency of coffee alone because the amount of intake at a time had not been analyzed in the FFQ in KNHANES before 2012. The largest difference between these previous studies and the present study was the calculation method of coffee consumption. Because we calculated the amount of coffee consumption using not only the frequency of intake, but also the amount of consumption at a time of intake, we could show a significant inverse association between coffee consumption and the prevalence of DM.

In the present study, the odds of DM were 0.92 in middle-aged women and 0.93 in middle-aged men, with 5-mL increments in daily powdered coffee consumption, despite adjustment for covariates. Most of previous studies analyzed the amount of coffee as a categorical variable and few studies analyzed the amount of coffee as a continuous variable. In a previous study, a 5-cup-per-day higher consumption was associated with an 8.8% reduction in 2-h glucose concentration [14]. Another study showed a 1 cup increase of coffee per day was associated with 0.16-units higher insulin sensitivity [15]. However, serving sizes for coffee and strength of the coffee brew can differ substantially within and between countries or individuals. In particular, the size of standard coffee cups is larger in the United States (≥250 mL) than in Europe (125–150 mL) [16]. We measured the amount of coffee using a teaspoon (5 mL) unit of powdered coffee. Therefore, it might be more accurate to calculate the amount of coffee consumption measured using a teaspoon or mL of powdered coffee, as delineated in the present study.

To the best of our knowledge, this is the first study that shows a clear inverse association between the prevalence of DM and coffee consumption in Korea, regardless of the type of coffee consumed. In fact, an inverse association between coffee consumption and the risk of DM has been observed in studies conducted in the United States, Europe, and Asia [4,12]. Consistent with these findings, several studies conducted in Asia showed that coffee consumption was inversely associated with the incidence of DM [17,18,19]. In fact, two studies performed in Korea showed an inverse association between coffee consumption and the risk of DM. However, they had some limitations. In one study, although the authors showed an inverse association between coffee and the risk of DM in total subjects, the association was not observed either in men or women. Furthermore, a linear trend was not observed [20]. The other study showed that the risk of high fasting blood glucose decreased as coffee intake increased. However, the exact amount or frequency of coffee consumption was not expressed according to the tertile grouping. In addition, only black coffee was considered in the study [21], in contrast to the present study, which included all types of coffee.

Coffee consumption by Korean adults increased 23.1% between 2009 and 2014, and most of the growth was observed for regular coffee. Although the pattern of consumption of coffee has changed, instant coffee still holds the highest proportion of the coffee market share [2]. Because instant coffee contains sugar and powdered creamers, many Korean coffee drinkers consume sugar and powdered creamers together. Furthermore, in the present study, subjects who consumed more coffee were more likely to be smokers, consume higher amounts of alcohol, report greater total energy intake, and have higher levels of blood cholesterol. In fact, higher coffee consumption has generally been associated with a less healthy lifestyle [4]. Therefore, the true association between coffee and diabetes risk might be stronger than observed. Previous studies showed that several components of coffee may ameliorate the risk of DM by affecting glucose regulation. These components include the effects of chlorogenic acid on glucose-6-phosphatase, the antioxidant activity of polyphenols on α-glucosidase, and the effects of caffeine on insulin secretion [22].

Given that aging is closely linked to the development of DM, it is of interest to distinguish potential differences in the association between coffee consumption and incidence of DM in younger versus older individuals. The association of coffee intake with DM incidence was not influenced substantially by mean age (≤50 and >50 years) [5]. However, the relationship between the prevalence of DM and coffee consumption was not significant in the young adult groups of the present study. The low prevalence of DM in the young adult groups might make the association less significant. In addition, it might take years to reduce the incidence of DM by usual coffee consumption. Therefore, it is more difficult to assume habitual coffee consumption from our cross-sectional data in young adult groups than in middle-aged adult groups. A cross-sectional design may explain why the present study could not show a reduced prevalence of diabetes in young adult groups. Further longitudinal studies conducted among subjects divided by age groups are needed.

To our knowledge, this is the first study to show a clear inverse relationship between the prevalence of DM and coffee intake using an accurate unit of measurement of powdered coffee, regardless of the type of coffee consumed. Although this study provided important results, it has some limitations according to the cross-sectional design. First, the cross sectional design limited any attempt to determine the causal directions of the identified associations. Second, the diagnosis of DM, including self-report, might elicit differential misclassification bias. Subjects’ knowledge of their DM status influenced how they reported coffee consumption. However, because DM was also objectively measured by the level of fasting glucose or HbA1c, differential misclassification of the outcome by level of the exposure was most unlikely. Third, an inverse relationship could not be accurately assessed in young adult groups. Fourth, the type of coffee consumed, as well as the level of caffeine, were not analyzed according to the SQFFQ used in KNHANES. Because the way coffee is brewed impacts the antioxidants and coffee oils produced, different types of coffee and different ways of consuming it would impact DM development. Therefore, a reduced level of DM prevalence, according to the coffee intake in this study, might not be easily extrapolated to other countries where coffee consumers drink different types of coffee beverages.

## 5. Conclusions

In conclusion, we found that a higher level of coffee consumption was associated with a lower prevalence of DM in Korean middle-aged adults. Adjustment for covariates did not modify the association between coffee intake and diabetes risk.

## Figures and Tables

**Table 1:** Baseline characteristics.

	Young Adult (Age 19–39)		Middle-Aged Adult (Age 40–64)		
	Women (*N* = 5,579,373)	Men (*N* = 6,130,535)	Women (*N* = 7,416,029)	Men (*N* = 7,119,478)	*p*
Mean Age (years)	29.38 ± 0.15 ^a^	29.51 ± 0.16 ^a^	51.02 ± 0.12 ^b^	50.48 ± 0.14 ^c^	<0.0001
Body Weight (kg)	56.66 ± 0.20 ^a^	73.56 ± 0.29 ^b^	58.61 ± 0.14 ^c^	71.02 ± 0.20 ^d^	<0.0001
BMI (kg/m^2^)	21.80 ± 0.07 ^a^	24.16 ± 0.09 ^b^	23.72 ± 0.06 ^c^	24.49 ± 0.06 ^d^	<0.0001
Current Smoker	411,539 (7)	2,555,421 (42)	301,593 (4)	2,858,968 (40)	<0.0001
Heavy Drinker	763,917 (14)	1,706,586 (28)	714,970 (10)	2,851,558 (40)	<0.0001
Physically Active	2,240,542 (41)	2,969,281 (49)	2,801,378 (38)	2,531,374 (36)	<0.0001
Income Level					<0.0001
Low Income	369,342 (7)	490,542 (8)	786,214 (11)	529,770 (7)	
Mid-Low Income	1,432,383 (26)	1,488,747 (24)	1,777,991 (24)	1,574,359 (22)	
Mid-High Income	1,917,837 (35)	2,102,974 (34)	2,193,064 (30)	2,243,656 (32)	
High Income	1,821,332 (33)	2,042,590 (33)	2,633,432 (36)	2,723,137 (39)	
Education Level					<0.0001
High School or Under	2,331,315 (42)	2,997,834 (50)	5,414,623 (74)	4,052,194 (58)	
College or Higher	3,203,207 (58)	3,057,928 (50)	1,943,892 (26)	2,972,194 (42)	
Systolic BP (mmHg)	105.03 ± 0.2 ^a^	115.15 ± 0.28 ^b^	116.24 ± 0.28 ^c^	120.62 ± 0.3 ^d^	<0.0001
Diastolic BP (mmHg)	69.84 ± 0.18 ^a^	76.48 ± 0.24 ^b^	75.47 ± 0.17 ^c^	80.84 ± 0.2 ^d^	<0.0001
Fasting Glucose (mg/dL)	90.25 ± 0.35 ^a^	92.5 ± 0.34 ^b^	98.57 ± 0.39 ^c^	104.13 ± 0.46 ^d^	<0.0001
HbA1c (%)	5.38 ± 0.01 ^a^	5.42 ± 0.01 ^b^	5.74 ± 0.01 ^c^	5.84 ± 0.02 ^d^	<0.0001
Hypertension	257,519 (5%)	1,318,652 (22%)	241,172 (33%)	3,340,347 (47%)	<0.0001
Hypertriglyceridemia	486,991 (9)	1,901,180 (31)	1,768,660 (24)	3,245,247 (46)	<0.0001
Reduced HDL	1,597,322 (29)	1,126,280 (18)	3,103,717 (42)	1,926,039 (27)	<0.0001
Total Energy Intake (kcal/day)	1966.4 ± 16.34 ^a^	2607.5 ± 22.22 ^b^	1729.2 ± 9.99 ^c^	2274.4 ± 15.26 ^d^	<0.0001

Qualitative data were expressed as counts and percentages, and qualitative variables were expressed as the means ± standard errors (SEs). BMI: body mass index; current smoker: currently smoking with a smoking history of ≥100 cigarettes in a lifetime; heavy drinker: drinking ≥2 times per week; physically active: walking ≥5 days in a week and ≥30 min in a day ≥10 min at a time; BP: blood pressure; HbA1c: hemoglobin A1c; hypertension: either a self-reported history of hypertension diagnosis and current usage of antihypertensive drugs, or a systolic BP of ≥130 mm Hg or a diastolic BP of ≥85 mm Hg; hypertriglyceridemia: triglyceride of ≥150 mg/dL; reduced HDL: HDL <40 mg/dL for men and <50 mg/dL for women. The *p* value was based on ANOVA for continuous variables and chi-square tests for categorical variables. Same superscript letters indicate no significant difference (*p* > 0.05) based on vertical pairwise comparisons with Bonferroni adjustment.

**Table 2:** Pattern of coffee intake among Korean adults.

	Young Adult (Age 19–39)		Middle-Aged Adult (Age 40–64)		
	Women (*N* = 5,579,373)	Men (*N* = 6,130,535)	Women (*N* = 7,416,029)	Men (*N* = 7,119,478)	*p*
**Mean Consumption Frequency/Week (tsp)**					
	8.01 ± 0.18 ^a^	10.4 ± 0.26 ^b^	10.68 ± 0.14 ^b^	15.12 ± 0.21 ^c^	<0.0001
**Consumption Amount/Intake**					
1tsp	2,377,591 (43%)	314,370 (51%)	5,334,981 (72%)	5,682,974 (80%)	<0.0001
2tsp	784,604 (14%)	832,930 (14%)	749,002 (10%)	520,995 (7%)	
3tsp or more	1,471,631 (26%)	1,244,819 (20%)	520,955 (7%)	427,880 (6%)	
**Consumption Amount/Day (tsp)**					
	1.97 ± 0.05 ^a^	2.24 ± 0.07 ^b^	1.97 ± 0.03 ^a^	2.72 ± 0.05 ^c^	<0.0001

Qualitative data were expressed as counts and percentages, and qualitative variables were expressed as means ± standard errors (SEs). tsp: teaspoon. The *p*-values were based on ANOVA for continuous variables and chi-square tests for categorical variables. The same superscript letters indicate no significant difference (*p* > 0.05) based on vertical pairwise comparisons with Bonferroni adjustment.

**Table 3:** Association between coffee consumption frequency per day and amount of coffee intake at a time.

Young Adult (Age 19–39)				Middle-Aged Adult (Age 40–64)			
Women (*N* = 5,579,373)		Men (*N* = 6,130,535)		Women (*N* = 7,416,029)		Men (*N* = 7,119,478)	
β	*p*	β	*p*	β	*p*	β	*p*
−0.4158	<0.001	−0.3485	<0.001	−0.4095	<0.001	−0.3212	<0.001

β: standardized coefficient of linear regression analyses.

**Table 4:** Association between the amount of daily coffee consumption and other covariates.

	Young Adult (Age 19–39)				Middle-Aged Adult (Age 40–64)			
	Women (*N* = 5,579,373)		Men (*N* = 6,130,535)		Women (*N* = 7,416,029)		Men (*N* = 7,119,478)	
	β	*p*	β	*p*	β	*p*	β	*p*
Mean age (years)	0.054	<0.0001	0.127	<0.0001	−0.061	<0.0001	−0.056	<0.0001
Body weight (kg)	0.009	0.108	0.015	0.008	0.009	0.034	0.008	0.08
BMI (kg/m^2^)	0.016	0.281	0.046	0.012	−0.002	0.821	0.008	0.569
Current smoker	1.225	<0.0001	1.347	<0.0001	1.352	<0.0001	1.236	<0.0001
Frequent alcohol consumption	0.690	<0.0001	0.524	0.001	0.574	<0.0001	0.049	0.66
Physically active	0.267	<0.0001	−0.221	0.148	−0.149	0.019	−0.041	0.699
Income level								
Low income	ref		ref		ref		ref	
Mid-low income	−0.104	0.952	0.478	0.033	0.207	0.053	0.535	0.016
Mid-high income	0.314	0.063	0.859	<0.0001	0.288	0.005	0.550	0.005
High income	0.627	0.001	1.034	<0.0001	0.560	<0.0001	0.606	0.001
Education level								
High school or under	ref		ref		ref		ref	
College or more	0.366	0.001	0.641	<0.0001	0.646	<0.0001	0.202	0.06
Fasting glucose (mg/dL)	0.002	0.486	0.008	0.104	−0.002	0.115	−0.004	0.021
HbA1c (%)	0.036	0.663	0.449	0.008	−0.146	<0.0001	−0.066	0.245
Total cholesterol (mg/dL)	0.007	<0.0001	0.009	<0.0001	0.001	0.238	0.005	<0.0001
Triglyceride (mg/dL)	0.001	0.2	0.001	0.004	−0.001	<0.0001	0.001	0.716
HDL (mg/dL)	0.009	0.104	−0.021	0.002	0.014	<0.0001	−0.015	<0.0001
Total energy intake (kcal/day)	0.000	0.147	0.000	<0.0001	0.000	<0.0001	0.000	<0.0001

β: standardized coefficient of linear regression analyses. BMI: body mass index; BP: blood pressure; HbA1c: hemoglobin A1c; HDL: high density lipoprotein. Current smoker: currently smoking with a smoking history of ≥100 cigarettes in a lifetime; heavy drinker: drinking ≥2 times per week; physically active: walking ≥5 days in a week and ≥30 min in a day with ≥10 min at a time. Values in bold indicate statistically significant values.

**Table 5:** Risk of prevalent diabetes according to the amount of coffee consumption after adjustment for covariates.

	Young Adult (Age 19–39)				Middle-Aged Adult (Age 40–64)			
	Women (*N* = 5,579,373)		Men (*N* = 6,130,535)		Women (*N* = 7,416,029)		Men (*N* = 7,119,478)	
	OR (95% CI)	*p*	OR (95% CI)	*p*	OR (95% CI)	*p*	OR (95% CI)	*p*
DM								
Model 1	0.861 (0.716–1.004)	0.079	0.977 (0.880–1.075)	0.653	0.918 (0.851–0.986)	0.023	0.934 (0.880–0.989)	0.022
Model 2	0.874 (0.727–1.021)	0.117	0.977 (0.882–1.071)	0.631	0.929 (0.861–0.997)	0.048	0.939 (0.883–0.994)	0.038
High fasting glucose								
Model 1	0.941 (0.874–1.009)	0.098	0.985 (0.941–1.029)	0.510	0.946 (0.908–0.984)	0.007	0.961 (0.930–0.993)	0.018
Model 2	0.948 (0.880–1.016)	0.147	0.976 (0.930–1.022)	0.316	0.952 (0.914–0.990)	0.018	0.958 (0.927–0.990)	0.011

DM: either fasting plasma glucose ≥126 mg/dL, current use of antidiabetic medication, history of previous diabetes, or glycosylated hemoglobin (HbA1c) ≥6.5%; high fasting glucose: either fasting glucose of ≥100 mg/dL or the presence of previously diagnosed diabetes. OR: odds ratio; CI: confidence interval. Values in bold indicate statistically significant values for multivariate logistic regression analysis. Model 1 adjusted for age (continuous), body mass index (continuous), smoking status (current smoker or not), and alcohol consumption status (drinking ≥2 times per week or <2 times per week). Model 2 adjusted for age, BMI, smoking status, alcohol consumption status, physical activity (physically active or not), income (four quartiles), education (high school or under and college or higher), and daily energy intake (continuous).
